# AuNPs@MIL-101 (Cr) as a SERS-Active Substrate for Sensitive Detection of VOCs

**DOI:** 10.3389/fbioe.2022.921693

**Published:** 2022-06-20

**Authors:** Dan Xie, Ruimeng Wang, Jinghao Fu, Zhongxing Zhao, Min Li

**Affiliations:** ^1^ CAS Key Laboratory for Biomedical Effects of Nanomaterials and Nanosafety, Institute of High Energy Physics, Chinese Academy of Sciences, Beijing, China; ^2^ Guangxi Key Laboratory of Agro-Environment and Agro-Product Safety, School of Chemistry and Chemical Engineering, Guangxi University, Nanning, China

**Keywords:** metal–organic framework (MOF), Gold nanoparticles (AuNPs), AuNPs@MIL-101(Cr), surface-enhanced Raman scattering (SERS), volatile organic compounds (VOCs)

## Abstract

Surface-enhanced Raman scattering (SERS) is an important and powerful analytical technique in chemical and biochemical analyses. Metal–organic frameworks (MOFs) can effectively capture volatile organic compounds (VOCs) with high adsorption capacity and fast kinetics, and the local surface plasmon resonance characteristics of gold nanoparticles can quickly and effectively distinguish different VOCs by SERS. Combining both, we designed a novel SERS substrate based on embedding gold nanoparticles (AuNPs) within MIL-101(Cr) for the recognition of various VOCs in the gaseous phase. Occupying of AuNPs inside MIL-101(Cr) increased the micropore-specific surface area of AuNPs@MIL-101(Cr), which enabled AuNPs@MIL-101(Cr) to absorb more toluene molecules and consequently realized its high detection sensitivity. The detection limits for toluene, 4-ethylbenzaldehyde, and formaldehyde were down to 6, 5, and 75, ppm respectively. Moreover, this substrate could be used for detecting different VOCs simultaneously. Finally, we discussed the enhancement of AuNPs outside and inside MIL-101(Cr) on the Raman signal.

## Introduction

Volatile organic compounds (VOCs) are major pollutants in our environment, and most are toxic to humans at high concentrations, making sensitive detection of VOC of high importance (Srivastava, 2003; Guo et al., 2004; Lai et al., 2020). VOC detection has been applied to monitor agricultural products, environment, food, beverages, drugs, etc. (Demeestere et al., 2007; Hurot et al., 2019; Ziyaina et al., 2019). The VOCs present in human-exhaled gases, blood, or urine may be closely related to some diseases and thus have prognostic and diagnostic values (Banday et al., 2011; Kumar et al., 2013; Tripathi et al., 2016). Current techniques for VOC detection include gas chromatography–mass spectrometry (GC–MS) (Bernier et al., 2000; Mirzaei et al., 2018), gas chromatography–ion mobility spectrometry (GC–IMS) (Allers et al., 2016), photoionization detector (PID) (Rezende et al., 2019), metal oxide sensor (MOS) (Elmi et al., 2008), optical sensors (Lin et al., 2019; Thongsai et al., 2019; Yang et al., 2019), or resistive gas sensors (Mirzaei et al., 2018). These methods have been highly successful but also have drawbacks. For example, GC–MS is commonly used for VOC detection due to its high sensitivity, accuracy, repeatability, and stability but possesses disadvantages of high cost, complicated operation, and long analysis time (Lubes and Goodarzi, 2018). The metal oxide sensor (MOS) is another common instrument for VOC detection, but it cannot simultaneously recognize different VOCs (Zampolli et al., 2007). The disadvantage of optical sensors for VOC detection is their complicated operation and high cost (Hsieh and Yao, 2018).

Due to their large pore size, large internal surface area, and active metal sites, MOFs are suitable for the study of catalysis, separation, gas storage, biomedical applications, and proton conduction. Meanwhile surface-enhanced Raman scattering (SERS) provides fingerprint molecular information for detection at the single-molecule level. Combining the high vapor adsorption capacity of metal–organic frameworks and the quick and effective VOC distinguishment of the SERS technique, high sensitive detection on VOCs has been achieved in a few reports to date. For example, using a zeolite imidazole MOF (ZIF) skeleton with Ag nanocubes as a platform (Ag @ ZIF), VOC-sensing by SERS to ppm levels was demonstrated (Koh et al., 2018). In another work, a thin film of ZIFs was grown on the surface of gold nanospheres. Then, a Raman active probe p-aminothiophenol (4-ATP) was modified on the gold nanospheres to recognize gaseous aldehydes and finally realized a detection limit of ppb (parts per billion) (Qiao et al., 2018). Recently, our group has proved that the MOF MIL-100 (Fe) platform can simultaneously detect different gases with ppm even to ppb detection limits (Fu et al., 2020).

Here, we fabricated a novel VOC detection system by embedding gold nanoparticles (AuNPs) inside MIL-101 (Cr) through an *in situ* HAuCl_4_ reduction approach. Combining the enhancement of the “hotspots” generated by AuNPs and the significant enrichment of MOF, gaseous analytes of interest are effectively detected. We showed that this system could detect toluene and 4-ethylbenzaldehyde down to 6 and 5 ppm, respectively.

## Experimental Section

### Materials

Chromium (III) nitrate [Cr (NO_3_)_3_·9H_2_O], 1,4-benzene dicarboxylic acid (H_2_BDC), and hydrofluoric acid (HF) were purchased from Aladdin Industrial Co. Ltd (Shanghai, China), and sodium citrate was obtained from Alfa Aesar. Toluene, chloroform, and formaldehyde were bought from Beijing Chemical Works.

### Instrumentation

Morphologies and microstructures of these prepared samples were carried out by using a scanning electron microscope (SEM, Hitachi S-3400N type). The crystal structure of the sample was analyzed by X-ray diffraction (XRD, SmartLab diffractometer, Japan) at a scan rate of 2°/min and a monochromatic X-ray beam with nickel-filtered Cu Kα radiation at 30 kV. Transmission electron microscopy (TEM) was performed on a JEM-2100 Plus transmission electron microscope at an acceleration voltage of 200 kV. Raman spectra were obtained on a DXR SmartRaman spectrometer (Thermo Fisher, 780 nm, 40 mW, and 10 μm diameter focal spot laser excitation, 15 s integration time, room temperature).

### Preparation of MIL-101 (Cr) and AuNPs@MIL-101 (Cr)

1) MIL-101 (Cr) was synthesized as follows: A 14.4 ml amount of aqueous solution containing Cr (NO_3_)_3_·9H_2_O (5 mmol), hydrofluoric acid (5 mmol), and H_2_BDC (5 mmol) was added to a hydrothermal autoclave reactor, which reacted at 220°C for 8 h. The green product and crystallized H_2_BDC byproduct were obtained after natural cooling. The product was purified by washing with DMF and ethanol (4,000 rpm, 15 min). The purified light green product was dried at 150°C under vacuum for further experiments.

2) AuNPs@MIL-101 (Cr) was prepared *via* a solution impregnation strategy. Briefly, 50 mg of MIL-101 (Cr) was suspended in 30 ml of 0.1% (w/v) HAuCl_4_ aqueous solution and was kept under continuous stirring at 45°C for 2.5 h. Then, the solution was heated to vigorous boiling, followed by a quick injection of 220 μl of 10% (w/v) sodium citrate and was further stirred for another 40 min at boiling temperature. After cooling, the AuNPs@MIL-101 (Cr) products were collected and purified by centrifugation (4,000 rpm, 10 min) 3 times.

### VOC Detection

HAuCl_4_ adsorbed and grew gradually in the cavity of MIL-101 (Cr) *via* an *in situ* reduction strategy to form AuNPs@MIL-101 (Cr) which was employed as the SERS substrate for VOC sensing. In detail, 20 µl (10 mg/ml) of MIL-101 (Cr) or 10 µl of AuNPs@MIL-101 (Cr) was first dropped onto a quartz container and dried in an oven for the preparation of the SERS substrate. Then, the substrate was exposed to a volatile solution in a 25-L sealed container for 15 min to allow both the evaporation of the volatile solution and its adsorption on MOF. The Raman spectra of VOCs were collected under 780-nm laser excitation.

The gold colloid was prepared according to the literature (Bastús et al., 2011), with the diameter of the AuNPs ca. as 30 nm. The as-prepared gold colloid was then mixed with MIL-101 (Cr) at a 1:1 ratio (v/v) for the preparation of the SERS-active substrate. After drying, the substrate was exposed to a volatile solution in a 25-L sealed container for 15 min. The Raman spectra of VOCs were collected under 780-nm laser excitation.

## Results and Discussion

**SCHEME 1:**
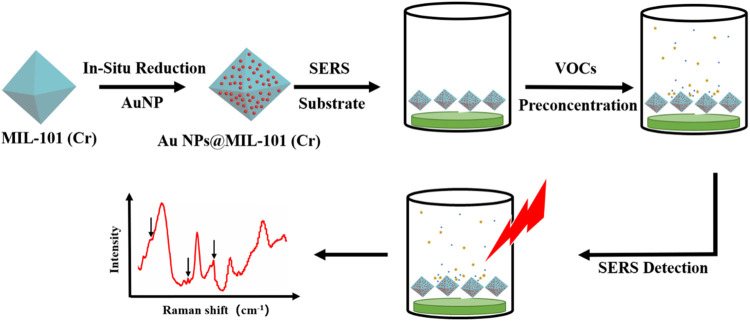
Schematic illustration of the use of AuNPs@MIL-101 (Cr) to detect volatile organic compounds (VOCs) by SERS.

As shown in Scheme 1, AuNPs@MIL-101 (Cr) was synthesized and considered as the SERS substrate for different VOCs’ detection. In order to prove that MIL-101 (Cr) has a high adsorption capacity for VOCs, we first used MIL-101 (Cr) as the SERS substrate to detect toluene and chloroform. As shown in Supplementary Figure S1A, the distinctive peaks of toluene at 1,001 cm^−1^ (ν_C = C_) and 785 cm^−1^ (δ_C = C_) became higher and higher with increasing concentrations of toluene in the container, constituting our VOC assay. The MIL-101 (Cr) platform, without AuNPs, could realize toluene sensing down to 83 ppm *via* SERS. Other VOCs such as chloroform could also be detected (see the distinctive peaks of chloroform at 667 cm^−1^ (ν_CCl_) in Supplementary Figure S1B). According to our previous work (Fu et al., 2020), VOCs preferred to adsorb into the structure of MIL-100(Fe) *via* π–π or coordination interactions. Similarly, pure MIL-101 (Cr) could follow the same rule. In other words, benzene compounds tended to interact with the aromatic ligand through π–π interaction, while small polar molecules were likely to adsorb to the MOF *via* Cr-heteroatomic bonds. Since VOCs could be detected via SERS using bare MOF material, we aimed to include noble metal nanoparticles into the MOFs to further improve their performance.

**FIGURE 1:**
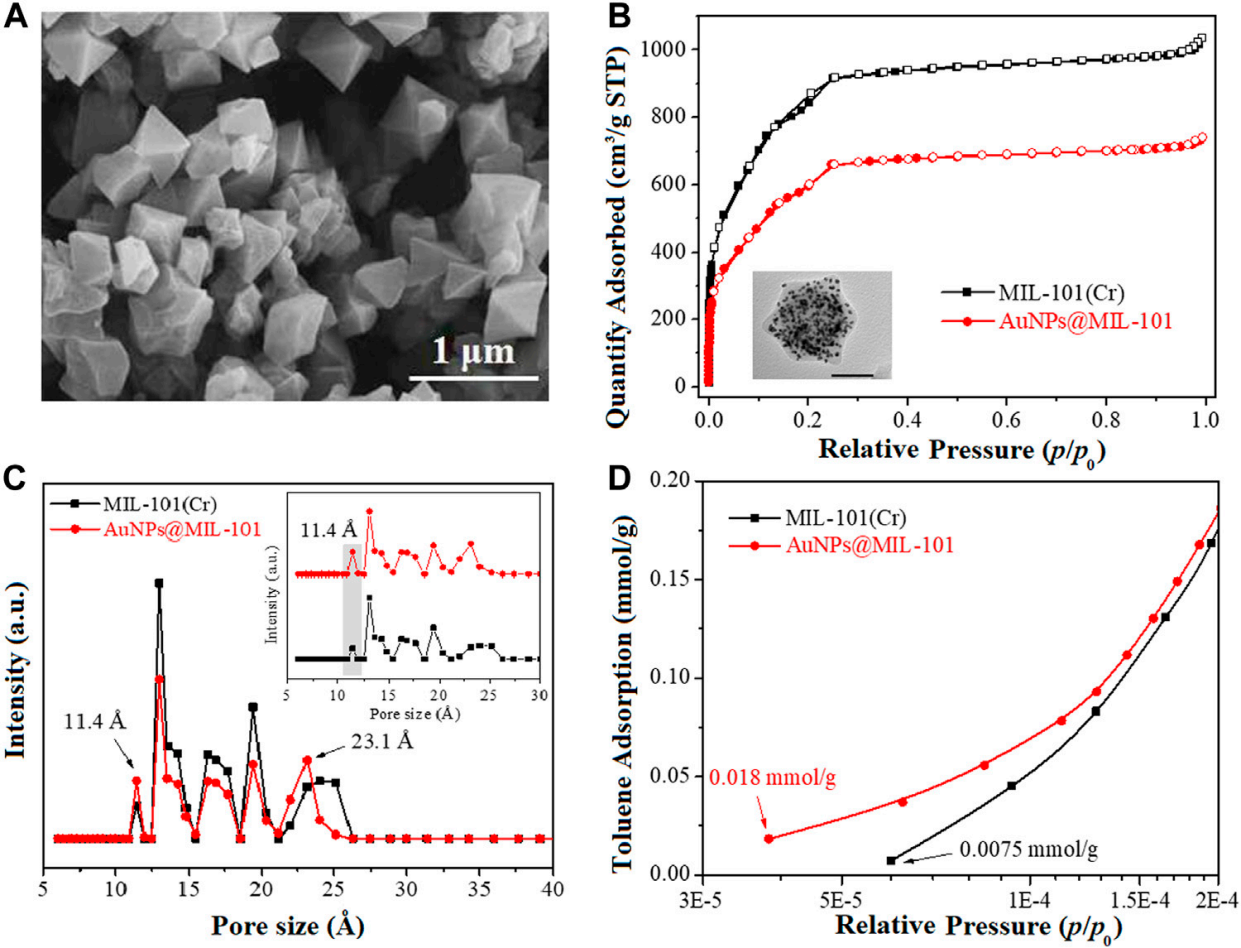
**(A)** SEM image, **(B)** N_2_ ad-/desorption isotherms, **(C)** pore size distribution of MIL-101 and AuNPs@MIL-101 (Cr), and **(D)** toluene adsorption isotherms of MIL-101 (Cr) and AuNPs@MIL-101 (Cr) at 298 K (*P/P*_*0*_ = 3.0 × 10^−5^–2.0 × 10^−4^).

A SEM image of the MOF material MIL-101 (Cr) is shown in Figure 1A. It exhibits a typical octahedral morphology with a smooth crystal surface and a particle size of about 0.65 μm, consistent with the previously reported work (Zhu et al., 2017; Zhao et al., 2020). Supplementary Figure S2A shows an N_2_ adsorption/desorption isotherm and the aperture structure of the pristine MIL-101 (Cr). The isotherm was type-I in nature (Zhu et al., 2017; Wang et al., 2021). In the range of *P/P*_*0*_ = 0–0.1, the adsorption capacity of the material for N_2_ increased rapidly, suggesting a typical microporous structure (Zhang et al., 2015). A second stage of micropore adsorption occurred in the N_2_ isotherm adsorption before *P/P*_*0*_ = 0.3, which was mainly due to the existence of two different pore sizes in the microporous windows in the MOFs structures (F´erey et al., 2005). Later, the N_2_ adsorption isotherm exhibited a distinct upward thrust at *P/P*_*0*_ > 0.9, implying the classic mesoporous adsorption. This indicated that the original MIL-101 (Cr) had a micro-/mesoporous structure.

Supplementary Figure S2B shows the PXRD pattern of MIL-101 (Cr), featuring strong diffraction peaks at 2*θ* = 2.9°, 3.4°, 5.3°, 8.5°, and 9.2°. These corresponded to the diffractions from the crystal planes (311), (511), (531), (882), and (911), respectively. Those were the typical diffraction peaks for MIL-101 (Cr) (F´erey et al., 2005; Ma et al., 2019), indicating the successful synthesis of highly crystalline MIL-101 (Cr).

To investigate the structural stability of MIL-101 (Cr), thermal gravity analysis (TGA) and differential thermal gravity (DTG) were carried out under a N_2_ atmosphere, and the resulting curves are shown in Supplementary Figure S2C. From the TGA curve, it could be seen that the mass loss percentage of MIL-101 (Cr) was only 71.1%, owing to its good structural stability. Meanwhile, the DTG curve showed three distinct peaks from 30 to 800°C, proving once again that the original MIL-101 (Cr) had good structural stability.

In order to illustrate the change of the structural properties of AuNPs@MIL-101 (Cr) after encapsulation of AuNPs, the N_2_ ad/desorption isotherms and pore size distributions of MIL-101 (Cr), as well as AuNPs@MIL-101 (Cr) are compared in Figures 1B,C. It could be seen that both samples were featured in Type-I isotherms (Zhu et al., 2017; Wang et al., 2021). Compared to MIL-101 (Cr), the N_2_ adsorption isotherms of AuNPs@MIL-101 (Cr) decreased significantly, indicative of a reduced specific surface area due to the encapsulation of AuNPs. Furthermore, there was no distinct upward trend at *P/P*_*0*_ > 0.9 in the N_2_ adsorption isotherms of AuNPs@MIL-101 (Cr), suggesting that the mesoporous volume decreased. Correspondingly, the pore size distribution of MIL-101 (Cr) and AuNPs@MIL-101 (Cr) was studied via the density functional theory (DFT) model in Figure 1C. Both samples were predicted to have a micro/mesoporous structure, and their main pore sizes were both concentrated in 10.9∼25 Å. However, it could be seen that the micro and mesoporous structures of AuNPs@MIL-101 changed remarkably in the DFT model due to the encapsulation of AuNPs. Compared with MIL-101 (Cr), the pore volume of AuNPs@MIL-101 (Cr) decreased, owing to the encapsulation of AuNPs which blocked parts of the pores. Notably, the proportion of pore size with 11.4 Å increased for AuNPs@MIL-101 (Cr), while the mesoporous pore size reduced to 23.1 Å. The increase of the micropore proportion of AuNPs@MIL-101 (Cr) at 11.4 Å might be beneficial to the adsorption of toluene at ultra-low pressure, owing to the smaller kinetic diameter of toluene molecules (∼6.8 Å) than 11.4 Å.

The pore structural parameters of MIL-101 (Cr) and AuNPs@MIL-101 (Cr) are listed in Supplementary Table S1. MIL-101 (Cr) had a specific surface area of 3,078 m^2^/g with a high total pore volume (1.60 cm^3^/g, V_t_), and the ratios of S_micro_/S_BET_ and V_micro_/V_t_ were 0.88 and 0.78, respectively. After AuNP encapsulation, the specific surface area and total pore volume of AuNPs@MIL-101 (Cr) reduced to 2,347 m^2^/g and 1.10 cm^3^/g due to the occupation of AuNPs in the MOF channels. The contribution of the micropores’ specific surface area and pore volume slightly increased to 0.91 and 0.80, respectively, higher than 0.88 and 0.78 for the original MIL-101 (Cr). This could be attributed to the encapsulation of AuNPs into the MOF channels, which changed the larger pores into smaller ones, making it more conducive to the adsorption of toluene by AuNPs@MIL-101 (Cr) at low pressure compared with MIL-101 (Cr).

To assist the understanding of the mechanism of Raman enhancement on AuNPs@MIL-101 (Cr), the toluene adsorption isotherms of MIL-101 (Cr) and AuNPs@MIL-101 (Cr) at 298 K were determined, as shown in Figure 1D and Supplementary Figure S3. Supplementary Figure S3 shows that the toluene adsorption isotherms of both samples were type-I in the range of *P/P*_*0*_ = 1.0 × 10^−5^—1.0, indicating a typical microporous adsorption process (Wang et al., 2021). It could be seen that MIL-101 (Cr) had a high saturated adsorption capacity for toluene (12.8 mmol/g). Compared with MIL-101 (Cr), the saturated adsorption capacity of toluene on AuNPs@MIL-101 (Cr) was reduced to 9.7 mmol/g due to its decreased specific surface area. However, Figure 1D indicates that the adsorption capacity of AuNPs@MIL-101 (Cr) for toluene was stronger than that of MIL-101 (Cr) in the range of *P/P*_*0*_ = 3.0 × 10^−5^—2.0 × 10^−4^. It could be seen that the initial toluene adsorption content of AuNPs@MIL-101 (Cr) was 0.018 mmol/g, much better than that of MIL-101 (Cr) (0.0075 mmol/g). More importantly, the starting point pressure of toluene adsorption on AuNPs@MIL-101 (Cr) was less than *P/P*_*0*_ = 4.0 × 10^−5^ which was lower than that of MIL-101 (Cr) (*P/P*_*0*_ = 6.0 × 10^−5^). Thus, under the conditions of ultra-low vapor pressures of toluene, AuNPs@MIL-101 (Cr) might adsorb toluene molecules more easily compared to MIL-101 (Cr) *via* the smaller pore size of AuNPs@MIL-101 (Cr). This is the main reason which caused the toluene enhancement and realized the lower detection limit for AuNPs@MIL-101 (Cr) (Fu et al., 2020).

Furthermore, we designed a AuNPs@MIL-101 (Cr) platform and used to concentrate VOC molecules and generate the “hotspots” between Au nanoparticles, thus attempting to achieve a sensitive SERS detection of VOCs based on the fact that SERS enhancement is closely related to the size and amount of AuNPs (Hu et al., 2014; Hu et al., 2017). Thus, we explored various concentrations of the HAuCl_4_ aqueous solution and sodium citrate, respectively, for the preparation of AuNPs@MIL-101 (Cr) to obtain the embedded AuNPs with different sizes. As shown in Supplementary Figure S4, we synthesized AuNPs with different sizes by using 30 ml HAuCl_4_ aqueous solution with concentrations of 0.13, 0.1, and 0.06% (w/v), respectively, while keeping 220 μl of 10% (w/v) sodium citrate unchanged.

To investigate the SERS activity of AuNPs with different sizes and different particle densities in MIL-101 (Cr), the performance of the AuNPs@MIL-101 (Cr) platform was tested for toluene detection. The results showed that the sensing properties of this platform were not significantly affected by the large (35 nm), medium (17 nm), or small (13 nm)-sized AuNPs in MIL-101 (Cr). It should be noted that the signal-to-noise ratio for tracing VOC detection on AuNPs@MIL-101 (Cr) in the case of encapsulating small-sized AuNPs was observed to be rather weak (see both Supplementary Figures S4A, S5A) The large, medium, and small-sized AuNPs within MIL-101 (Cr) exhibited a detection limit for toluene at 13 ppm, 6 ppm, and 25 ppm, respectively, indicating that 30 ml of 0.1% (w/v) HAuCl_4_ aqueous solution could be considered as the better conditions.

We further synthesized AuNPs within MIL-101 (Cr) under different concentrations of sodium citrate [220 μl of 12% (w/v), 15% (w/v), and 20% (w/v)] when fixing the conditions of HAuCl_4_ aqueous solution (30 ml, 0.1% (w/v)). As shown in Supplementary Figure S5, large (27 nm)-, medium (20 nm)-, and small (13 nm)-sized AuNPs within MIL-101 (Cr) were synthesized *in situ* in MIL-101 (Cr), and the hybrid materials presented detection limits of 9 ppm, 17 ppm, and 34 ppm, respectively, for toluene sensing, as shown in Supplementary Figure S5. Comparing the sensing performance of AuNPs@MIL-101 (Cr) shown in both Supplementary Figures S4, S5, 30 ml of 0.1% (w/v) HAuCl_4_ aqueous solution and 220 μl of 10% (w/v) sodium citrate were considered as the optimized conditions to prepare AuNPs@MIL-101 (Cr) with the best performance.

**FIGURE 2:**
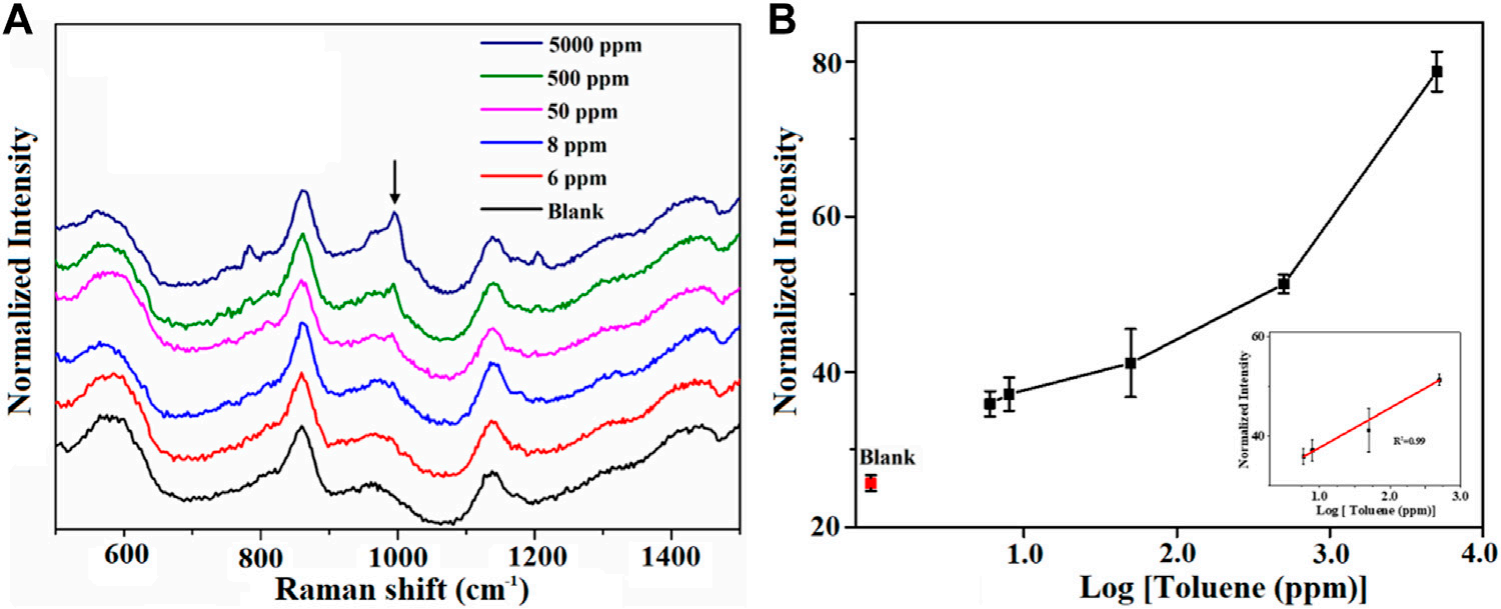
**(A)** Raman spectra of the AuNPs@MIL-101 (Cr) platform for detecting toluene at different concentrations. **(B)** Semi-log plot of their normalized Raman intensities as a function of toluene concentrations. The normalized intensities are based on the peak at 858 cm^−1^.

To further study the SERS properties of AuNPs@MIL-101 (Cr), different concentrations of toluene were carefully detected. As shown in Figure 2, the distinctive Raman peaks of toluene at 1,001 cm^−1^ (ν_C = C_) and 785 cm^−1^ (δ_C = C_) became higher and higher with increasing toluene concentration. The detection limit for toluene was demonstrated to be 6 ppm, which was much lower than that of bare MIL-101 (Cr) (83 ppm, Supplementary Figure S1) but similar to the bare MIL-100 (Fe), where the toluene detection limit was down to 2.5 ppm, as reported in our previous work (Fu et al., 2020). As shown from the results of the N_2_ adsorption isotherm and DFT simulation of the pore distribution, the contribution of the micropore specific surface area of AuNPs@MIL-101 (Cr) increases due to AuNPs occupying parts of the MOF channels, which enables AuNPs@MIL-101 (Cr) to absorb more toluene molecules and consequently realizes its detection with high sensitivity. As shown in Figures 2A,B, the linear relationship between their normalized Raman intensities and the log concentration of toluene (*R* ^2^ = 0.99) from 5 to 500 ppm was obtained, indicating an exponential relationship. The shape of the curve indicates again that the toluene adsorption kinetics behavior for AuNPs@MIL-101 (Cr) was a typical microporous adsorption process.

**FIGURE 3:**
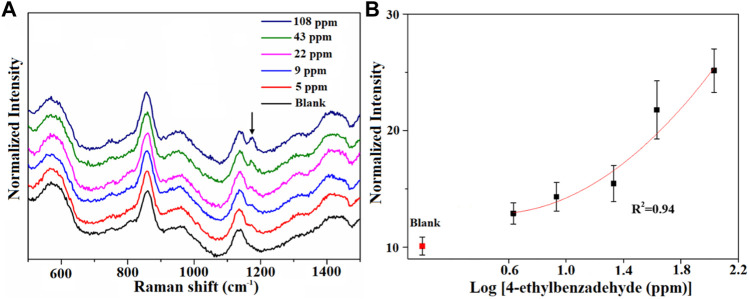
**(A)** Raman spectra of the AuNPs@MIL-101 (Cr) platform for detecting 4-ethylbenzaldehyde at different concentrations. **(B)** Semi-log plot of their normalized Raman intensities as a function of 4-ethylbenzadehyde concentrations. The normalized intensities are based on the peak at 858 cm^−1^.

Then, we assessed the performance of AuNPs@MIL-101 (Cr) for different VOCs’ detection. The conditions of the attempt of 4-ethylbenzaldehyde’s adsorption on AuNPs@MIL-101 (Cr) were set at 60°C and stayed for 40 min, owing to the relatively higher boiling point of 4-ethylbenzaldehyde. As shown in Figure 3, with increasing concentration, the intensity variation of the distinctive peaks of 4-ethylbenzadehyde at 1,174 cm^−1^ [δ_CH_ (benzene ring)] was observed. The detection limit for 4-ethylbenzadehyde was down to 5 ppm. Figure 3B shows the relationship of their normalized Raman intensity as a function of 4-ethylbenzadehyde concentrations.

As is known, formaldehyde is ubiquitous in our living environment, being emitted from newly built houses, and furnishings. It is seriously harmful to human health. Ultrasensitive detection of formaldehyde vapor is therefore very important. Thus, the AuNPs@MIL-101 (Cr) platform was also tried for formaldehyde detection. The distinctive peaks of formaldehyde at 485 cm^−1^ are shown clearly in Supplementary Figure S6. The detection limit of formaldehyde was determined to be 75 ppm.

**FIGURE 4:**
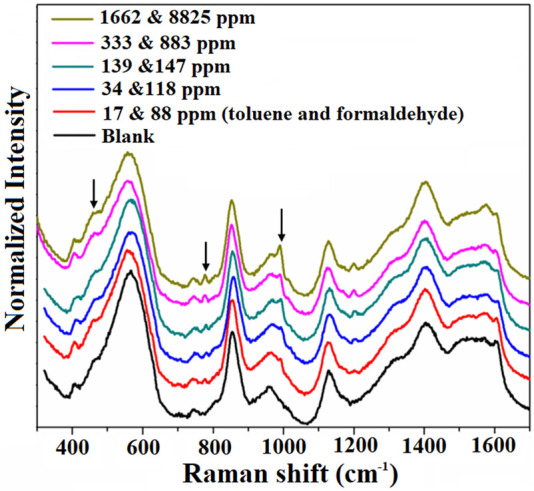
Raman spectra of the AuNPs@MIL-101 (Cr) platform for simultaneous detection of toluene and formaldehyde at different concentrations. The normalized intensities are based on the peak at 858 cm^−1^.

Finally, we investigated the multiplex-sensing properties of this AuNPs@MIL-101 (Cr) platform on the simultaneous detection of toluene and formaldehyde. As shown in Figure 4, the intensity of distinctive peaks of toluene and formaldehyde increases with the increasing VOC concentrations. The AuNPs@MIL-101 (Cr) platform could realize the detection limits of 17 and 88 ppm, respectively, for toluene and formaldehyde simultaneous sensing without pronounced cross-talk, as indicated in Figure 4.

**FIGURE 5:**
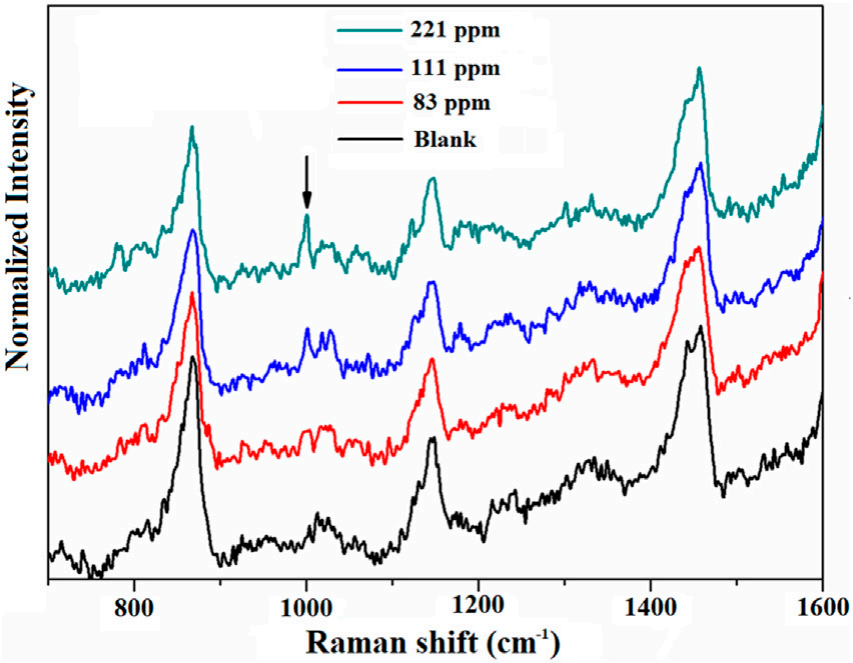
Raman spectra of MIL-101 (Cr) in combination with Au colloids at 1:1 ratio (v/v) for the detection of different concentrations of toluene.

Next, we investigated the contributions of AuNPs located both inside and outside MIL-101 (Cr) to the Raman enhancement. It should be noted that the amount of AuNPs on the surface of the MIL-101 (Cr) is very little; most of the AuNPs are present in the solution. To clarify the contribution of the AuNPs in the solution to the observed SERS signals, we performed an experiment in which MIL-101 (Cr) was combined with Au colloids in a 1:1 ratio (v/v) for the detection of toluene with different concentrations. The results are shown in Figure 5. The detection limit for toluene in this case was approximately 83 ppm, which is similar to pure MIL-101 (Cr) for toluene sensing. Therefore, the enhancement of the Raman signal comes mainly from the AuNPs inside but not outside the MIL-101 (Cr).

We also tested the performance of Au colloids in toluene sensing at 1,050 ppm. Supplementary Figure S7 shows that a distinctive peak of toluene at 1,001 cm^−1^ was observed after adsorption by the Au colloid. When the Au colloid was diluted twice, the peak could also be distinguished. However, no observable peak appeared for the case of five times-diluted Au colloids on toluene sensing at 1,050 ppm. These results proved that the sensing performance of the Au colloids on toluene was rather poor but could be greatly enhanced when encapsulated within the MOF.

## Conclusion

In conclusion, we demonstrated a sensitive platform involving the incorporation of AuNPs within MIL-101 (Cr) to detect VOCs at ppm levels by SERS. Using this platform, the detection limit for toluene could reach 6 ppm, and for formaldehyde, it could reach 76 ppm. Furthermore, the platform can simultaneously detect different VOCs at the ppm-level without obvious interferences/cross-talk. Two factors were found to be essential for the efficiency of the AuNPs@MIL-101 (Cr) VOC detection platform. First of all, the occupation of AuNPs in the channels inside MIL-101 (Cr) increases the micropores’ specific surface area of AuNPs@MIL-101 (Cr), which facilitated the adsorption of toluene on AuNPs@MIL-101 (Cr) and consequently increased its detection sensitivity. Second, a large number of AuNPs were incorporated into MIL-101 (Cr), which made a contribution to the SERS signal enhancement. The ability of AuNPs@MIL-101 (Cr) for multiplex sensing of VOCs and the gaseous odors with low Raman cross-section, together with its great modifiability and expandability, etc., will be of particular potential toward studying the recognition process of odors.

## Data Availability

The original contributions presented in the study are included in the article/Supplementary Material; further inquiries can be directed to the corresponding authors.
