# Supplementary figures and images for: AuNPs@MIL-101 (Cr) as a SERS-Active Substrate for Sensitive Detection of VOCs
Source: Front Bioeng Biotechnol. 2022 Jun 20;10:921693. doi: 10.3389/fbioe.2022.921693 (PMC9256292; doi:10.3389/fbioe.2022.921693)

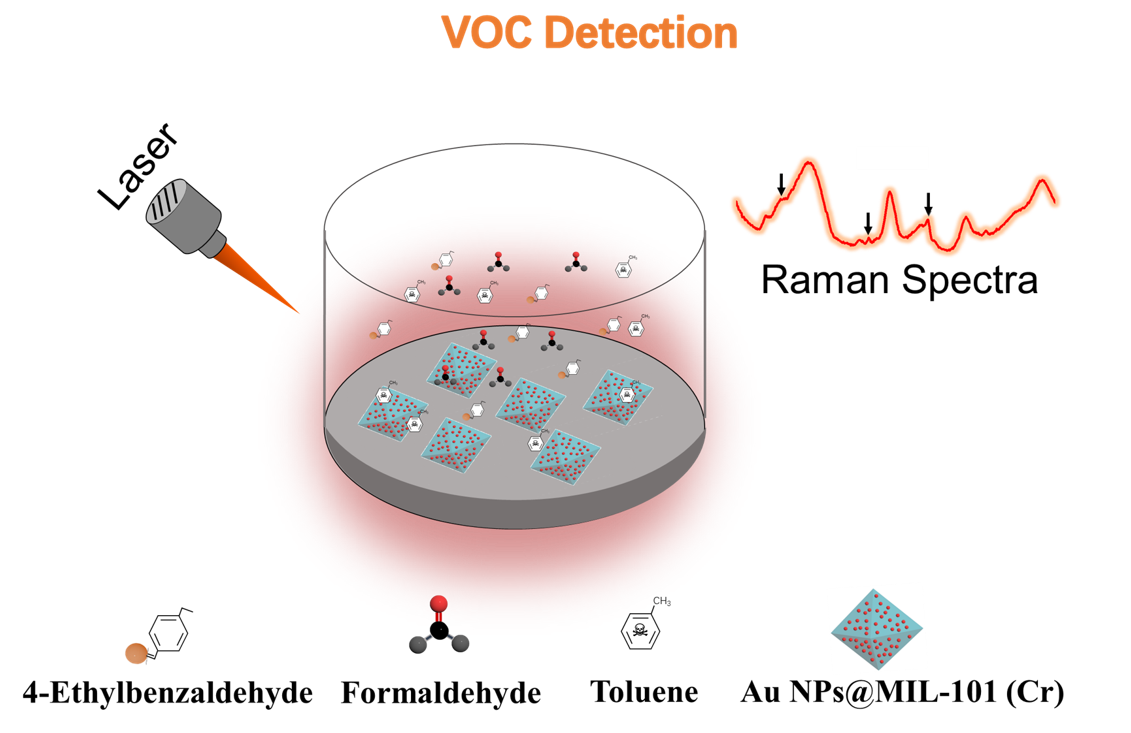

Supplement: Supplementary file 1 [file Image1.TIF]
