# Supplementary material for: AuNPs@MIL-101 (Cr) as a SERS-Active Substrate for Sensitive Detection of VOCs
Source: Front Bioeng Biotechnol. 2022 Jun 20;10:921693. doi: 10.3389/fbioe.2022.921693 (PMC9256292; doi:10.3389/fbioe.2022.921693)
Supplement: Supplementary file 2 [file DataSheet1.docx]

Supplementary Material


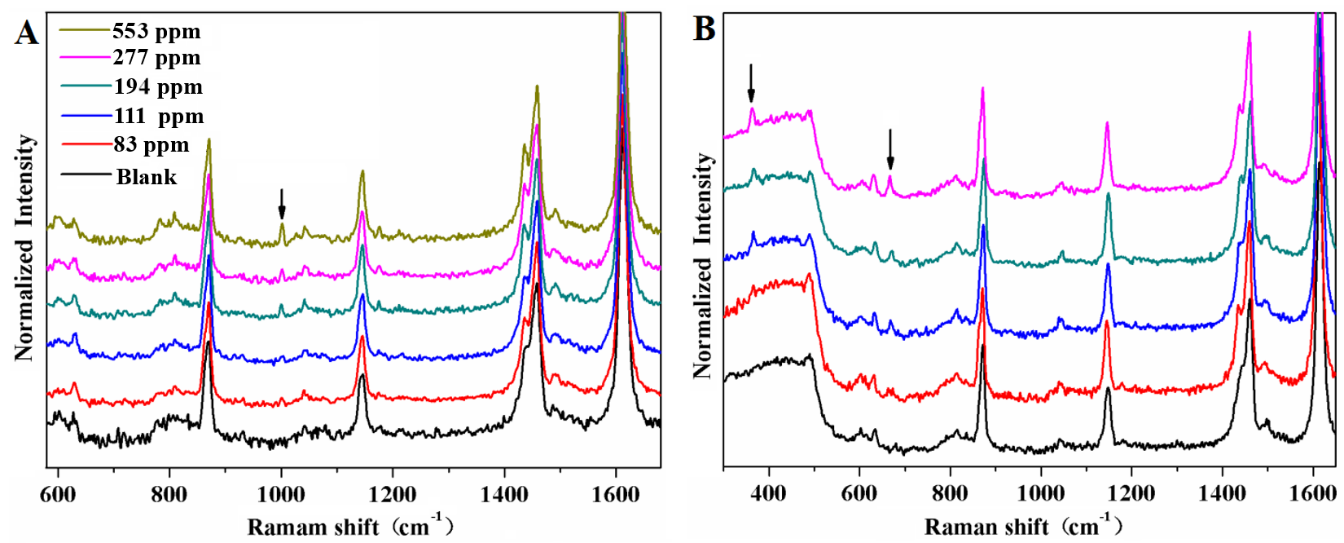


**FIGURE S1**. SERS spectra of MIL-101(Cr) detected different concentrations of toluene **(A)** and Chloroform **(B)**. The normalized intensities are based on the peak at 1611 cm^-1^.


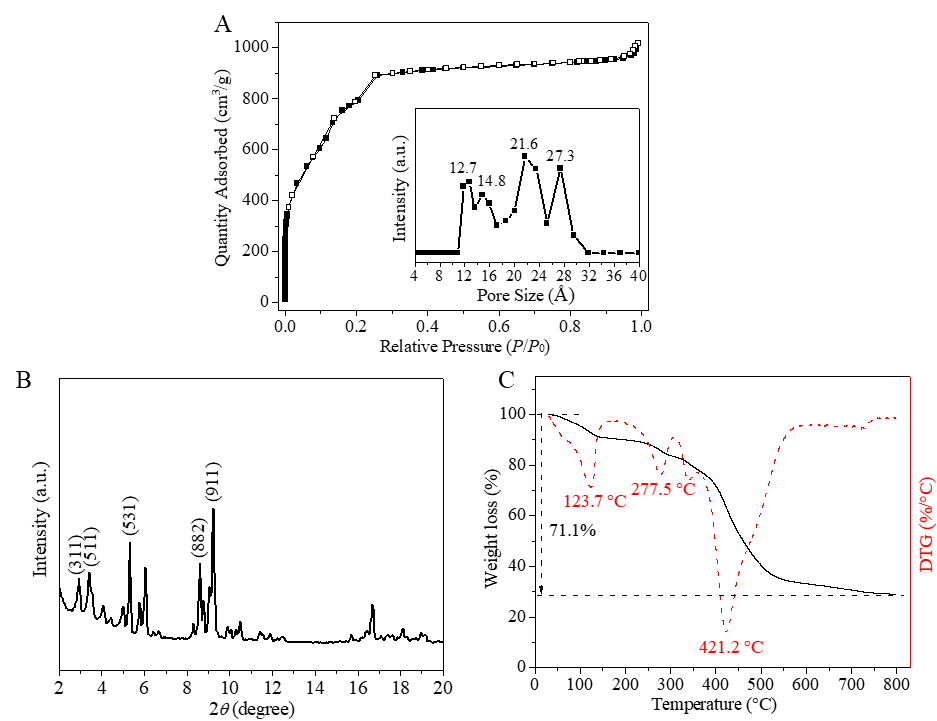


**FIGURE S2**. **(A)** N2 adsorption isotherm and pore size distribution of prinstine MIL-101(Cr) *via* density functional theory (DFT) models. Pore sizes of 21.6 Å and 27.3 Å are predicted to be present in relatively higher proportion compared to pore sizes of 12.7 Å and 14.8 Å in the sample. **(B)** PXRD pattern of pristine MIL-101(Cr). **(C)** TGA and DTG curves of pristine MIL-101(Cr). The first peak is due to desorption of surface adsorbed water at 123.7°C, showing the material has a strong hydrophilicity. After that, at 277.5°C, some functional groups like hydroxyl and carboxyl groups of the sample are destroyed, attributed to the desorption of water bound within the material. Finally, the sample exhibited a higher structural decomposition temperature at about 421.2°C, proving that the original MIL-101(Cr) has good structural stability.

**TABLE S1** Pore structure parameters of MIL-101(Cr) and AuNPs@MIL-101.

| **Sample** | S_BET_**^a^ *(m^2^/g)*** | S_micro_^b^ ***(m^2^/g)*** | ***S_meso_*^b^ *(m^2^/g)*** | S_micro_/S_BET_ | V_total_**^c^ *(cm^3^/g)*** | V_micro_**^d^ *(cm^3^/g)*** | V_meso_**^d^ *(cm^3^/g)*** | V_micro_/V_t_ |
| --- | --- | --- | --- | --- | --- | --- | --- | --- |
| **MIL-101(Cr)** | ***3078*** | ***2719*** | ***359*** | ***0.88*** | ***1.60*** | ***1.25*** | ***0.35*** | ***0.78*** |
| **AuNPs@** MIL-101(Cr) | ***2347*** | ***2132*** | ***215*** | ***0.91*** | ***1.10*** | ***0.88*** | ***0.22*** | ***0.80*** |

^a^ *S*_BET_ is the surface area of BET;

^b^ *S*_micro_ and *S*_meso_ is the surface area of microporous and mesoporous structure, respectively;

^c^ *V*_total_ is the total volume;

^d^ *V*_micro_ and *V*_meso_ is microporous and mesoporous volume, respectively.

**FIGURE S3**. Toluene adsorption isotherms of MIL-101(Cr) and AuNPs@MIL-101(Cr) at 298 K (P/P_0_=1.0×10^-5^-1.0).


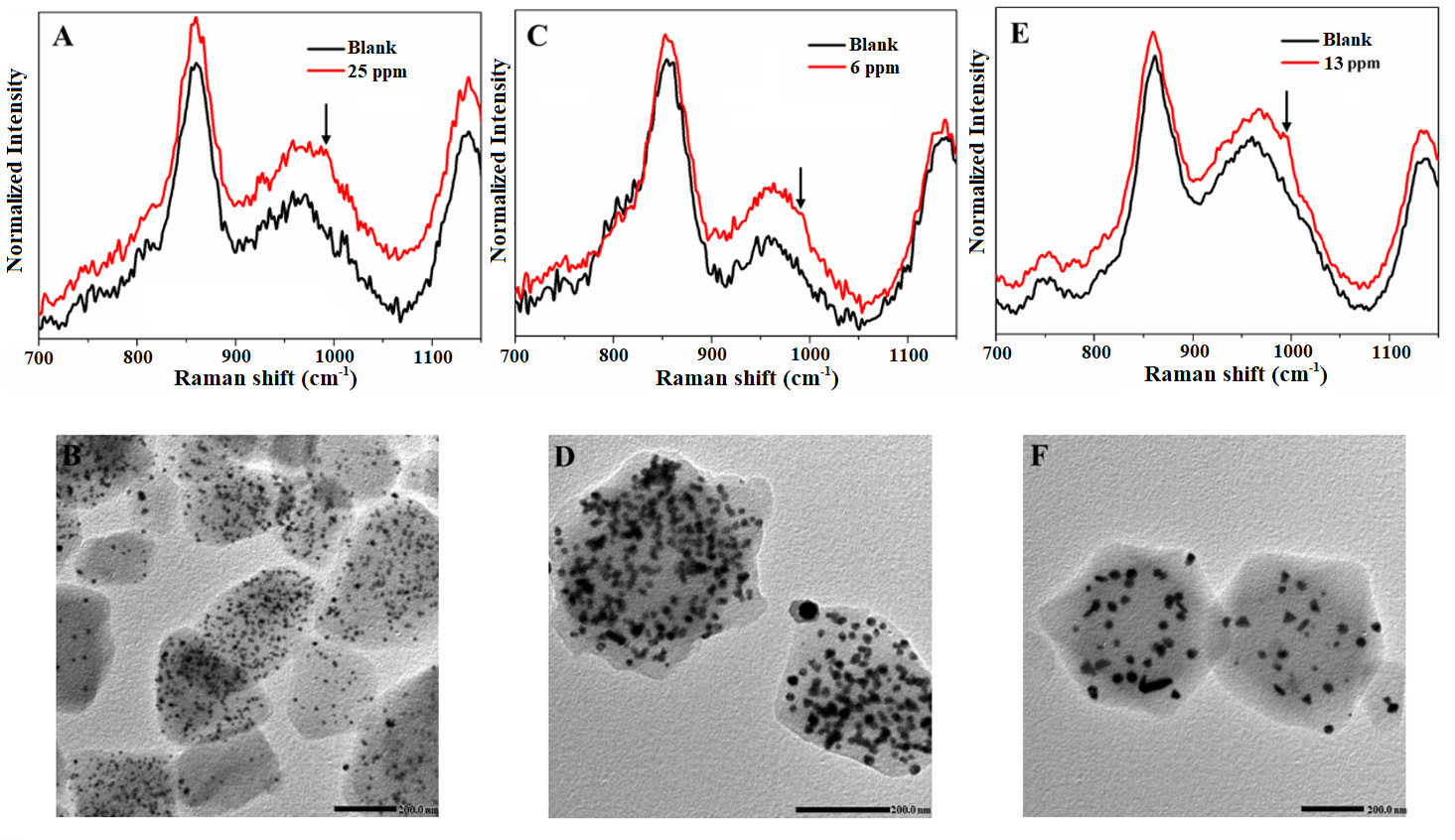


**FIGURE S4**. SERS spectra of small (13 nm) **(A)**, medium (17 nm) **(C)**, large (35 nm) **(E)** sized AuNPs within MIL-101(Cr) for toluene sensing at the detection limit respectively, and TEM images of small **(B)**, medium **(D)**, large **(F)** sized AuNP within MIL-101(Cr). The normalized intensities are based on the peak at 858 cm^-1^.


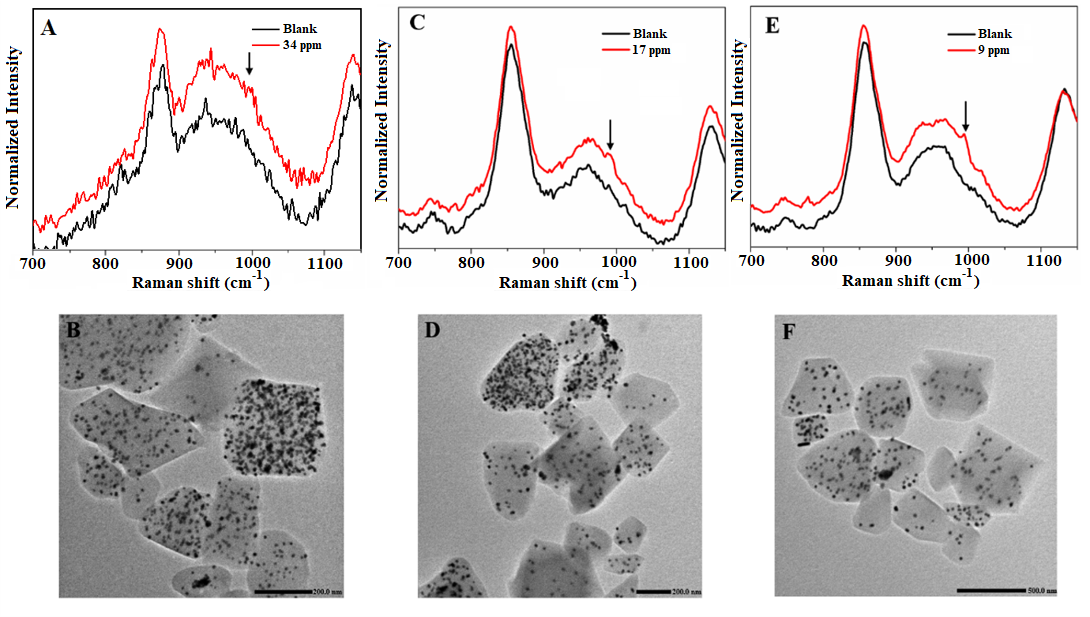


**FIGURE S5**. SERS spectra of small (13 nm) **(A)**, medium (20 nm) **(C)**, large **(E)** (27nm) sized AuNP within MIL-101(Cr) for toluene sensing at the detection limit respectively, and TEM images of small **(B)**, medium **(D)**, large **(F)** sized AuNP within MIL-101(Cr). The normalized intensities are based on the peak at 858 cm^-1^.


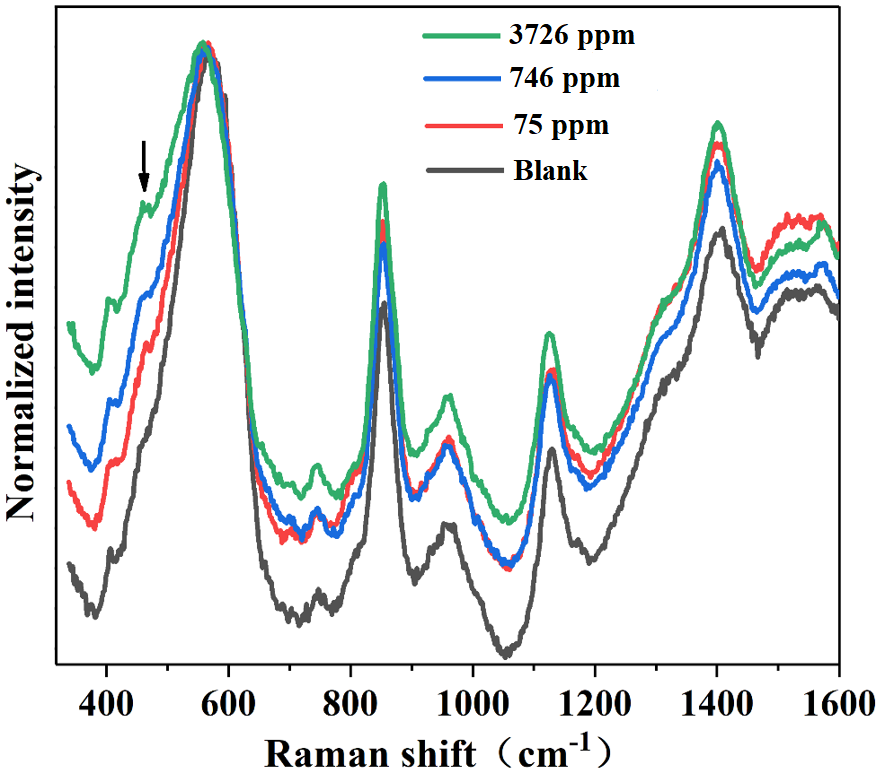


**FIGURE S6**. SERS spectra of AuNPs@MIL-101(Cr) for the detecting of formaldehyde with different concentrations. The normalized intensities are based on the peak at 858 cm^-1^.


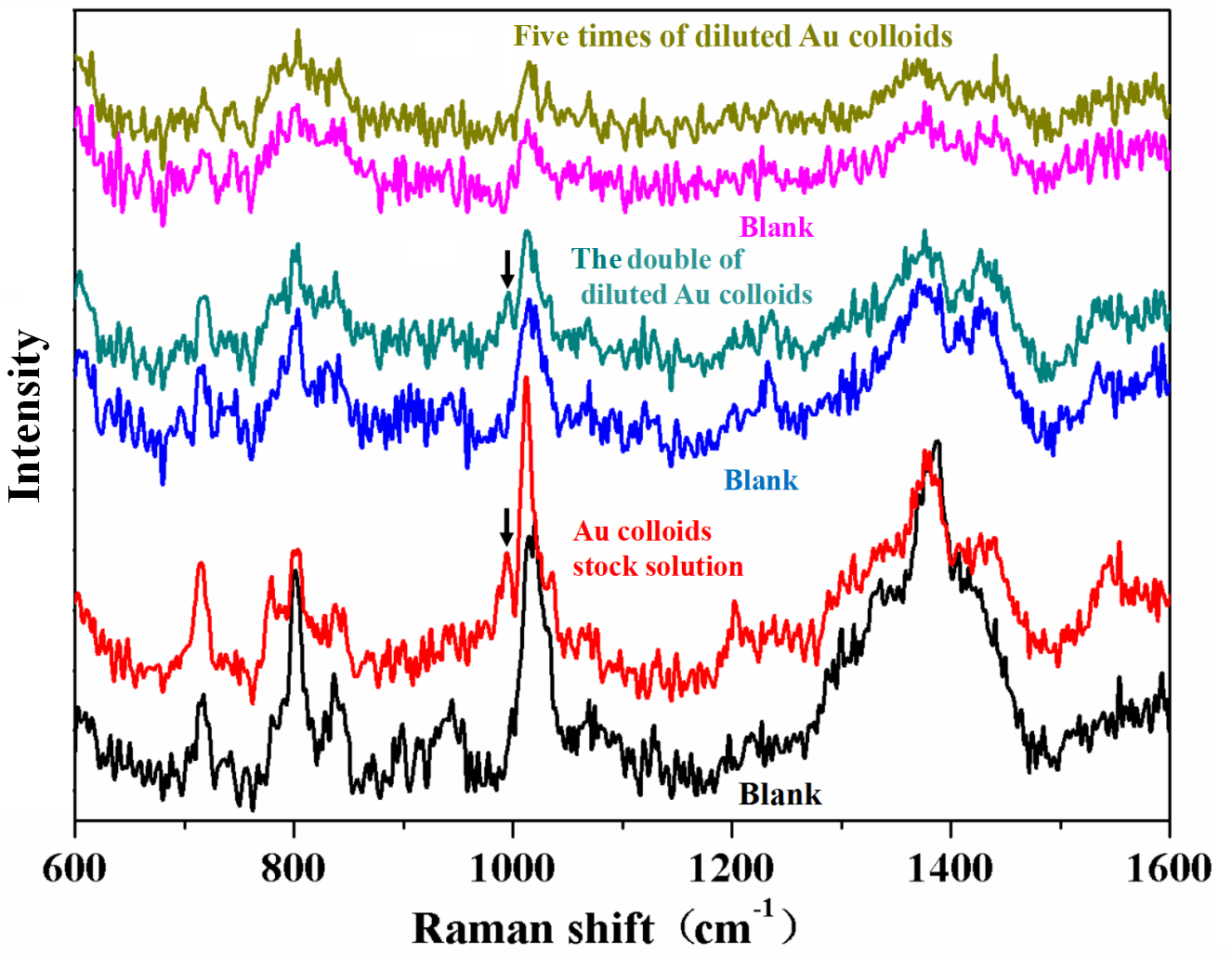


**FIGURE S7**. The detection performance of Au colloids with different dilution times on toluene at 1050 ppm.
